# How Did Awareness, Emotion, and Motivation Shape Behavior Toward COVID-19 in Tunisians?

**DOI:** 10.3389/fpubh.2021.771686

**Published:** 2021-12-31

**Authors:** Slim Masmoudi

**Affiliations:** College of Criminology, Naïf Arab University for Security Sciences (NAUSS), Riyadh, Saudi Arabia

**Keywords:** COVID-19, psychological distress, mental health, awareness, emotion, cognition, behavior change, AB-Survey

## Abstract

This study was conducted to assess the psychological distress in the general population of Tunisia during the pandemic of COVID-19 and examines how awareness of the disease, emotional experience, and motivation shaped the behavior toward this outbreak. The study covers 1492 Tunisian participants. Gender effects and age limits were studied in relation with the seriousness of the disease and lockdown impacts. The data were analyzed and interpreted using the chi-square test, ANOVA, path analysis (PA), and confirmatory factor analysis (CFA). We found a significant effect of gender and age on the awareness of the pandemic dangerousness, the attitude, and the commitment to respect the preventive measures. Women are significantly more aware and committed than men to respect preventive health measures. The 35–45 age group showed more awareness and dedication than the other age groups, while the 20–35 age group showed the most less promise. PA and CFA results showed how awareness, emotion, and motivation shaped behavior toward COVID-19. This study provides practical and relevant information on behavior change during a pandemic crisis, which can orient communication campaigns and help policymakers.

## Introduction

The Coronavirus Disease 2019 (COVID-19 or SARS-CoV-2) outbreak began in Wuhan, China (1), and quickly spread through the country and then around the world, causing the World Health Organization (WHO) to announce COVID-19 a pandemic on March 11, 2020 (2). Together with severe physical issues, the COVID-19 pandemic has caused intense anxiety and panic in most societies (3) and feelings of boredom, isolation, and frustration among those who have been isolated (4). Consequently, and as with previous epidemics and pandemics, the COVID-19 pandemic has triggered a wide variety of negative psychological responses in the general population, involving anxiety and depression (5–8).

Therefore, this study was conducted to assess the psychological distress in the general population of Tunisia during the COVID-19 outbreak and examine how awareness of the disease, emotional experience, and motivation shaped the behavior toward COVID-19 (9–12). Results will give some valuable insights to policymakers and interveners in the field. First, the general pandemic context in Tunisia and over the world is described. Then, the mental health challenges and emotional issues are analyzed. Next, the central role of perception and awareness is discussed. Afterwards, the gender effect and the risky aversion attitude are analyzed. Finally, the age effect and attitude toward the pandemic are discussed.

Early March saw the first cases of COVID-19 recorded in Tunisia. To combat the pandemic, which was wreaking havoc on the vulnerable health system, the government took security steps. The state of emergency was declared, accompanied by the closing of the border, limiting population movement to necessities such as grocery shopping and finding emergency medical services. Health messages were provided to Tunisian citizens regularly, emphasizing the importance of staying at home, wearing a face mask when outside, hand washing, and social distancing (13, 14). The effectiveness of governments' attempts to reduce the spread of the virus is influenced by public opinion. Several studies have been found to determine the public's attitude toward safety measures and the attitudes of particular occupational groups. In Saudi Arabia, the general population has a moderate attitude toward regulation and prevention measures. Respondents said that social distancing, handwashing, staying at home, and the following department of health guidelines would aid in controlling COVID-19's spread in their country (15). The Chinese government was confident in its ability to control the transmission of the COVID-19 pandemic and win the war against it (16). Malaysian government employees expressed optimism about the government's ability to control the COVID-19 pandemic. They were optimistic that COVID-19 would be contained and that since their government handles health problems so well, their country would be able to resolve them (17). Up to April 24, 2021, 12:50 GMT, Coronavirus Cases attain 146,348,573, with 3,102,348 deaths and 124,170,337 recovered cases. The world sanitary crisis continues to threaten lives, and its effective management needs to be informed on important psychological variables as awareness, emotion, motivation, and behavior change toward the pandemic.

From an epidemiological standpoint, widespread implementation of health-protective behavior, such as enhanced personal hygiene or social distancing, is typically successful in minimizing or slowing the spread of contagious respiratory diseases (18). However, when avoidance behaviors result in prejudice against subgroups of people or a lack of essential needs like food or prescription goods, they may have severe social and economic consequences (19). The question of whether the discovery of the coronavirus sparked a panic reaction, i.e., excessive public risk expectations and emotion-driven behaviors in light of available epidemiological evidence, prompted somewhat conflicting statements from prominent experts in the early stages of the COVID-19 epidemic. Dr. Michelle Dusart, the physician in the Saint-Pierre Hospital, strongly condemned the public's exaggerated paranoia and panic about the possibility of coronavirus infection. At the same time, Prof. Didier Raoult and Prof. Nassim Nicholas Taleb claimed that people's fear of such an emerging health danger was understandable. However, since the media tends to emphasize unusual behavior and knows so little about what people think about the hazard of coronavirus infection, it is impossible to say if they are excessively positive or cynical about the COVID-19 epidemic's long-term effects on public health.

COVID-19's unpredictability and ambiguity endanger not only people's physical health but also their mental wellbeing, especially in terms of emotions and comprehension, according to several studies. Disasters such as pandemics are said to trigger depression and complexity, which occurs because we may not be equipped to cope with such epidemics and their emotional consequences (20). In the nations, healthcare regulators and policymakers have enforced crises and shutdowns, which has had a negative impact on people's mental health, leading to an increase in anxiety, depression, and other mental illnesses. The COVID-19 crisis has raised fear and uncertainty, placing a strain on our scarce resources (20).

In such situations, people are more likely to experience negative emotions [e.g., irrational fear, anxiety, etc.; (21, 22)] and negative cognitive appraisal (23, 24) for self-protection, according to the Behavioral Immune System (BIS) theory (25). People develop avoidance habits [e.g., To avoid communication with people that have pneumonia-like signs; (26)] and strictly follow social standards (e.g., compliance) when they are faced with a possible disease danger. According to stress theory (27), public health crises and perceived danger theory induce more negative emotions and affect cognitive evaluation. When it comes to illness, these negative feelings hold people away from possible pathogens. The intensified anxiety and fear caused by the COVID-19 crisis put a strain on people's internal capacities. Decision-making issues, confusion, and nervous fatigue are among the consequences (20).

Lockdown also had a profound effect on the mental health of people. The sudden shutdown of the world economy left people jobless with nothing to do other than to stay locked up at home. This left people in an array of sadness and anxiety. Not being able to meet your loved ones and friends was already a big shock to people, but not setting foot out of the house left everyone in depression. Previous research has found that people under lockdown are more likely to develop stress, depression, emotional exhaustion, insomnia, and post-traumatic anxiety symptoms (28). Gender has been shown as a determinant factor in the way people cope with the lockdown (29).

On the other hand, long-term negative emotions can impair people's immunity and disrupt the balance of their usual biological systems (30). We can track psychological changes over time using emotional (e.g., negative emotions and positive emotions) and cognitive measures because psychological changes triggered by public health crises can be directly mirrored in emotions and cognitions [(22–24); e.g., social risk judgment and life satisfaction]. Meanwhile, individuals can retaliate to any disease if officials provide insufficient guidance, resulting in excessive avoidance and blind compliance (31). As a result, it is important to grasp the potential psychological effects of COVID-19 immediately.

Similarly, fear also plays a vital role in the psychological point of view of the individuals. A major psychological reaction to a health crisis is fear of a pandemic. Some recent research has concentrated on the negative effects of the global epidemic and social exclusion on mental health and psychological well-being at both the global and regional scales (32, 33). In these effects, the fear expresses itself in emotions, attitudes, actions, and on a psychological level can be illustrated, as it is activated both by the powerful proximity of the phobic stimuli and by the expectation of this future touch (34). Fear may have various outcomes, such as certain people doing desired acts due to their fear, while others do not. Another implication is that when people are afraid, they can respond in highly inappropriate forms (35). Fear is necessary for human survival because it is a component of the adaptive defense system for triggering behaviors to potentially dangerous events, rather than being a pathology in and of itself.

On the other hand, fear can raise anxiety and stress in healthy people during serious events like pandemics and escalate symptoms in people who already have mental disabilities, generating short to long-term psychological repercussions (36, 37). Broche-Pérez and his team (38) specified in their study that women are more likely to feel fear and anxiety and succumb to depression than men.

In addition to emotions' impact on behaviors and behavior change, perception and awareness play a central role. Generally speaking, risk perception and the awareness of the behaviors to be adopted and implemented shape behavior change and the attitude toward rules and measures. This was confirmed in smoking behavior (11), and in the protective behaviors against COVID-19 spread and similar future pandemics. A significant risk perception and awareness can lead to positive attitudes which are necessary to beat the pandemic. Nevertheless, Xu and Peng stated that the links between behavior and risk perception are unstable, and that “the evolving patterns of risk perception and responsive behavior over the course of an influenza pandemic are sensitive to how risk and behavior are defined and scoped” [(39), p. 1].

In addition, all the psychological traits have a strong connection with risk perception and the awareness about the important measures to be taken, compared to the ambiguity perception that does not affect the psychological traits. There is also a profound relationship between risk, benefits, and judgments. For instance, if an object is judged approvingly, then overall, it will be evaluated positively instead of negative evaluation. A recent study by Qian and his team (40) indicated that psychological and behavioral reactions were influenced by perceptions of morbidity, mortality, severity, and knowledge reliability. It was also found that a strong association exists between self-confidence and optimism among people and the lower risk of epidemic. The results indicate that irrespective of quarantine status, anxiety levels and preventative practices shifted quickly and significantly during the early stages of the outbreak (41, 42).

Regarding the relationship between gender and risk-aversion, our analysis confirms findings from previous literature that women are more risk-averse than men. From the research, it has been found that the hazard's quality has a significant impact on the perception of risk either exposure to the hazard is voluntary or in control, or its results are whether catastrophic, or its advantages are distributed fairly among those who can bear the risks. The studies suggest that men, compared to women, are usually found to be perceived the more eager to take risks (11, 43–45). A study recommended that the known risks are placed on higher-value by people than the unknown ones, i.e., uncertainty (46). It can be termed as ambiguity aversion. Its main function is to rationalize the equity-premium puzzle, also to analyze the different actions taken by different people in difficult circumstances. Women usually show higher ambiguity-aversion than men, but if the ambiguity increases, so then both men and women respond high (46, 47).

Regarding age effect on awareness and attitudes toward the pandemic, some studies has shown that the older the respondents, the better their attitude toward the disease (48). However, as the pandemic progressed older people tended to adopt mitigating personal behavioral changes more than younger people (49). Thus, two months after the pandemic started, older people showed more compliance with suggested measures and regulations including practicing quarantining, social distancing, and better hygiene. After the beginning of the pandemic, older people were less likely than younger people to adopt risky behaviors. In their study, Kim and Crimmins observed that “the change in risky behavior over time did not differ by age; but both younger and older people were more likely to engage in risky behaviors after two months” [(49), p. 1].

This study supposes that women are more risk-aversive, more aware and adopt less risky behaviors than men. They are supposed to show more compliance to preventive measures and more commitment than men. The 35–45 aged people are supposed to be more aware of the pandemic's seriousness than the youngest people and, consequently, more committed to respecting the preventive measures. Awareness and emotion are supposed to determine the specific committed behaviors and shape the general commitment.

## Method

### Participants

For this study, ethical approval has not been necessary since the data are properly anonymized and informed consent was obtained at the time of original data collection. The study was conducted in the Tunisian population. It was administrated from March 14 to March 25, 2020. It covered 1492 Tunisian participants aged 16 to 92 years old who could connect to the Internet and who were residents in Tunisia. Three hundred and twenty one men (21.52%) participated to the study with an average age of 34,31 (*SD* = 12,35), and 1,155 women (78.48%) participated with an average age of 31,32 (*SD* = 10,16). All participants were asked to check a dedicated box to confirm their consent to participate in the study. They have been informed that participation is completely anonymous, apart from certain demographic data.

The sample size was determined and constrained by the persons available to participate voluntary in the study through the provided form on the web and through the government Covid-19 website.

### Measure: AB-SURVEY

The study was based on the administration of the AB-SURVEY (Attitude-Behavior Survey) tool online (see Supplementary Material). This tool was specially conceived for the study and structured in five sections as follows: (1) Demographic section (five items), (2) Awareness/Perception section (two items), (3) Commitment/Behavior section (five items), (4) Emotion section (seven items), (5) Motivation/General Commitment section (three items). All the questions were formulated in French and Arabic since Tunisians are bilingual, and as we did not want the language to be a barrier. We used four different scales to get answers, according to the questions: Scale one (Very little, Little, Often, Always); Scale two (Very little, Little, A lot, Totally); Scale three (Yes, No); and Scale four (from 1 to 4, to measure the degree of emotions).

### Procedure

The AB-SURVEY form is distributed through the social media network and the government's official website, using the governmental information platform related to COVID-19. All participants were informed via the introduction of the form before starting that the participation is completely anonymous. They have to check the box confirming their consent to participate. Answering the questionnaire lasts no more than 7 min.

### Statistical Analysis

After receiving 1560 responses and after cleaning the data set (eliminating 60 participants for missing data and eight participants for providing inconsistent data such as an inconsistent age (e.g., 2 years), or a tendency to give the same answer to all the questions), we kept 1492 for analysis. SPSS Package V20 was used to perform Chi-square and ANOVA analysis, while LISREL V8.72 was used to make Path Analysis (PA) and Confirmatory Factor Analysis (CFA).

## Results

### Reliability of the AB-SURVEY

As shown in Table 1, the overall alpha Cronbach of the tool was 0.7. With acceptable alpha coefficient values for perception/awareness, behavior (specific commitment), and general commitment, this tool shows a good reliability.

**Table 1:** Reliability of the different components of the AB-SURVEY.

**Perception/Awareness**		**Behavior (Specific Commitment)**			**Motivation/General Commitment**		
**Evaluation** **of gravity**	**Evaluation of** **Speed of Spread**	**Hand** **Cleaning**	**No** **Touch**	**Social** **Distancing**	**Commitment**	**Behavioral** **Change**	**Optimism**
Alpha Cronbach = 0.71							
Alpha Cronbach = 0.713					Alpha Cronbach = 0.715		
Alpha Cronbach = 0.7							

### Confirmatory Factor Analysis

Confirmatory factor analysis was performed using maximum likelihood approximation with four factors (50) to see whether the latent model is confirmed or not. Several statistics were used to investigate the model's goodness of fit: Overall χ2 root means the square error of approximation (RMSEA), Akaike's information criterion (AIC), and comparative fit index (CFI). The AB-SURVEY CFA shows acceptable absolute and relative indices for the goodness of fit (Table 2). This allows us to consider with good confidence the confirmed model. The results show that the model presented an acceptable fit, with RMSEA ranging between 0.096 and 0.10.

**Table 2:** The goodness of fit statistics for AB-SURVEY.

**Absolute Fit indices**				**Relative Fit Indices**		
CMIN/DF	RMSEA	GFI	AGFI	CFI	NFI	TLI
9.155	0.096	0.91	0.86	0.87	0.86	0.882

**Figure 1:**
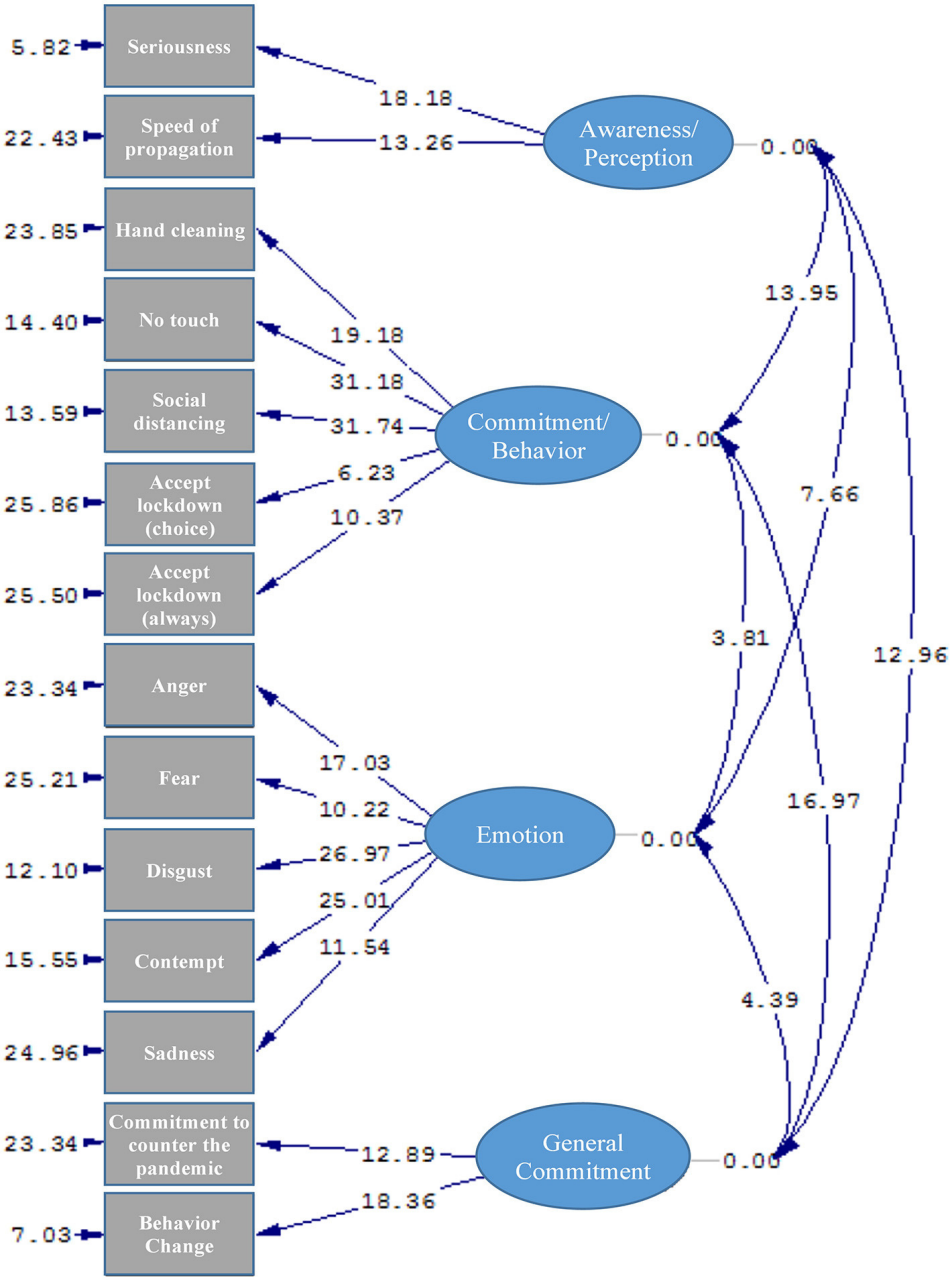
The resulting model (*T*-Values) from the CFA analysis (χ2 = 968.69; *df* = 71; *p* < 0.001; RMSEA = 0.096).

**Figure 2:**
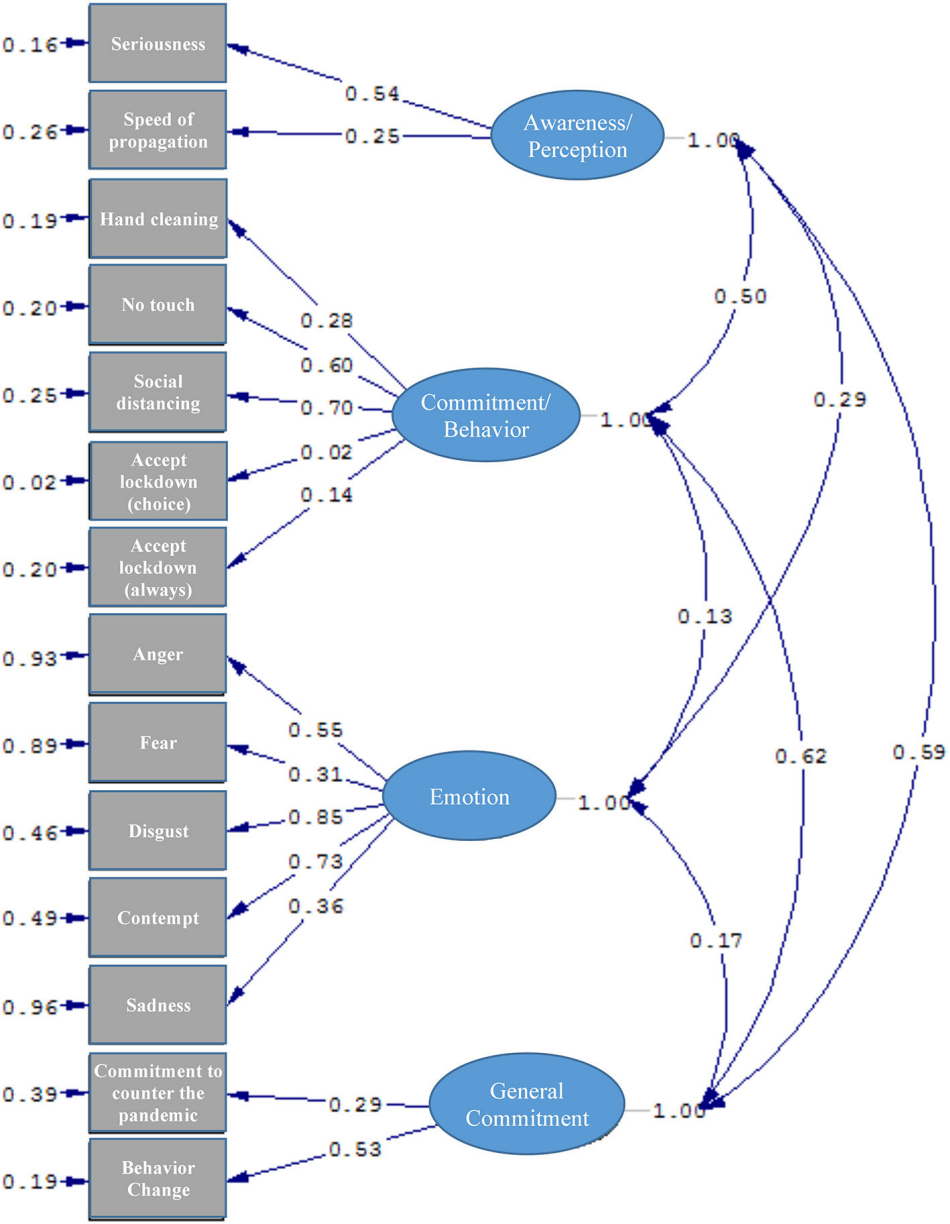
The resulting model (Estimates) from the CFA analysis (χ2 = 968.69; *df* = 71; *p* < 0.001; RMSEA = 0.096).

The model graph in Figures 1, 2 shows the estimated values between the four factors and their respective components.

### Path Analysis Out of the Different Models

Path analysis is an extension of the regression model, used to test the fit of the correlation matrix against two or more causal models compared by the researcher (51). Our hypotheses represented in the 6-path model are tested using SEM with Maximum Likelihood Estimation. results show that models 3 and 6 were supported while models 1, 2, 4, and 5 were not supported (Table 3). The RMSEA, CFI, GFI, and AGFI values showed that the path model had a somewhat good fit for the observed data for models 3 and 6.

**Table 3:** The goodness of fit statistics for the Path Analysis.

	**RMSEA**	**CFI**	**GFI**	**AGFI**
Model 1	0.31	0.56	0.94	0.63
Model 2	0.22	0.71	0.93	0.76
Model 3	0.076	0.98	1	0.97
Model 4	0.15	0.88	0.97	0.89
Model 5	0.31	0.57	0.94	0.63
Model 6	0.092	0.97	0.99	0.96

**Figure 3:**
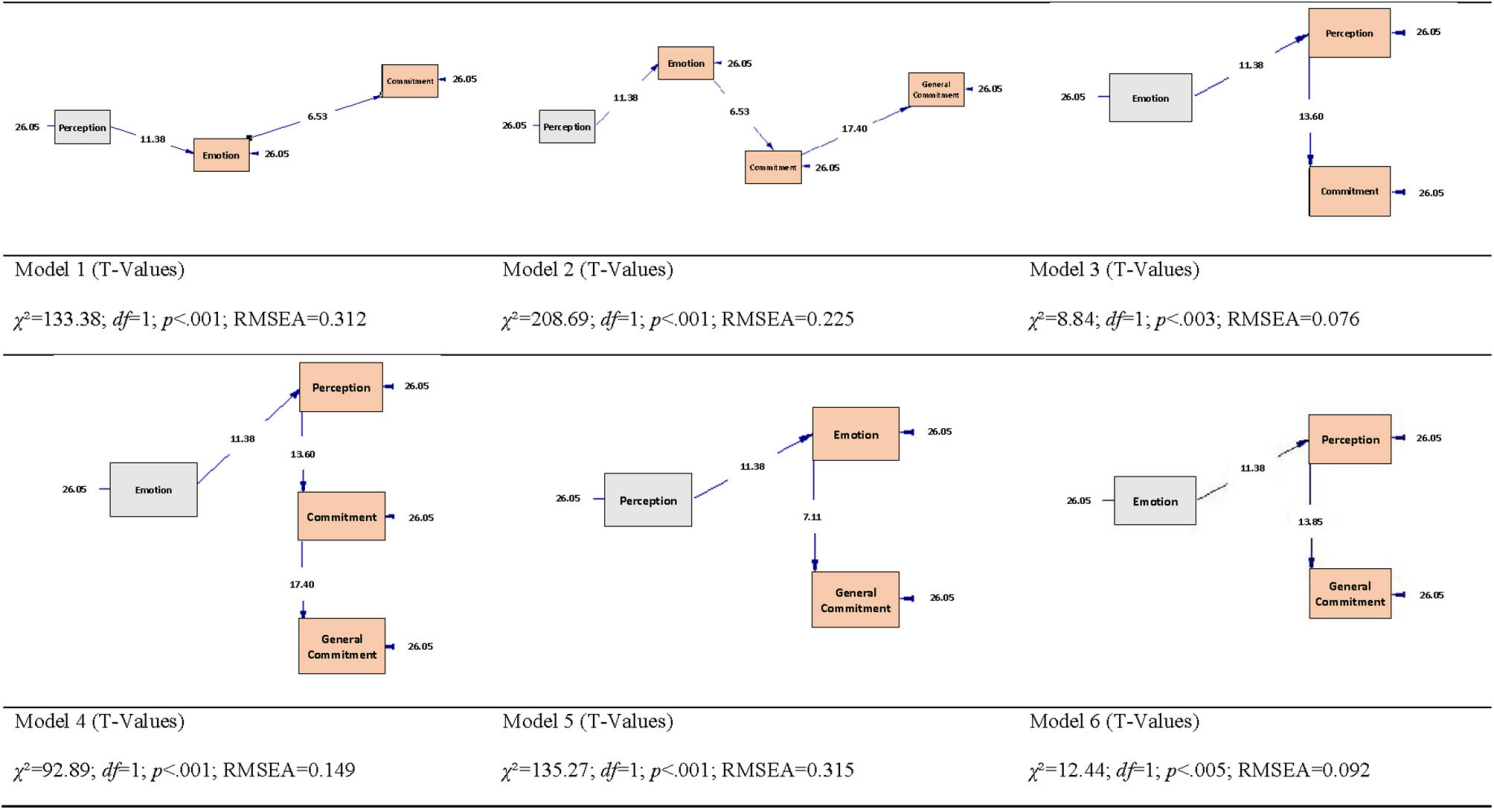
Graph models of Path Analysis from 1 to 6.

The path graphs (Figure 3) show how models 3 and 6 represent respectively the relationships between emotion, perception, and commitment (precautious behaviors), and emotion, perception and general commitment. Thus, emotion has an impact on commitment and general commitment through the modulation effect of perception. This would be a strong basis and justification for the importance to change perception to increase engagement. This could be achieved through communication and evidence-based information.

### Gender Effect: Women Are More Aware and Committed

The chi-square test of independence showed a significant relationship between gender and awareness of the necessity of the lockdown. Women are significantly more convinced about lockdown in any case: χ^2^ (1, 1492) = 3.97, *p* = 0.047; and about Lockdown in case of doubt about own infection: χ^2^ (1, 1492) = 5.94, *p* = 0.014.

The chi-square test of independence also showed a significant relationship between gender and awareness of the pandemic's dangerousness. Women are more aware of the dangerousness of the pandemic: χ^2^ (3, 1492) = 26.56, *p* = 0.007.

Seemingly, the chi-square test of independence was performed to examine the relationship between gender and the extent to which the preventive health measures were respected. Hand cleaning, touching other persons and social distancing are significantly related to gender, respectively χ^2^ (3, 1492) = 24.63, *p* = 0.02; χ^2^ (3, 1492) = 22.21, *p* = 0.05; χ^2^ (3, 1492) = 10.25, *p* = 0.02. The preventive health measures (hand cleaning, social distancing, and mask-wearing) are accomplished to minimize coronavirus transmission and are more respected by women than men.

### Emotion, Motivation, and Commitment According to Gender

A chi-square test of independence was performed to examine the relationship between gender and the extent to which the basic emotions were felt at the beginning of the lockdown. The relationship between these variables was significant, Fear: χ^2^ (3, 1492) = 66.576, *p* < 0.001; Sadness: χ^2^ (3, 1492) = 41.635, *p* < 0.001; Joy: χ^2^ (3, 1492) = 18.758, *p* < 0.001; Serenity: χ^2^ (3, 1492) = 34.435, *p* < 0.001. Women were more likely than men to feel fear and sadness and less than men to feel joy and serenity (Table 4).

**Table 4:** Percentages of women and men regarding the four degrees of fear, sadness, joy, and serenity (percentages).

**Degree**	**1**	**2**	**3**	**4**	**1**	**2**	**3**	**4**	**1**	**2**	**3**	**4**	**1**	**2**	**3**	**4**
	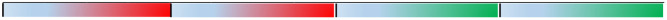															
**Fear**				**Sadness**				**Joy**				**Serenity**				
Women	9.9	21.0	36.1	33.1	11.2	20.3	29.8	38.7	90.2	5.3	2.0	2.5	64,9	17,1	10,8	7,3
Men	18.6	35.1	30.8	15.5	22.0	24.4	30.5	23.2	82.3	11.6	2.1	4.0	48,1	21,7	21,1	9,1
Together	11.8	24.1	34.9	29.2	13.5	21.2	30.0	35.3	88.5	6.7	2.0	2.8	60,9	18,2	13,2	7,7

*Dark color means high degree and clear color means low degree*.

When we considered the four-degree scale of emotions as an interval variable, the mean comparison between women and men led to significant differences regarding fear [*t*_(1492)_ = −6,931, *p* < 0.001], sadness [*t*_(1492)_ = −6,874, *p* < 0.001], joy [*t*_(1492)_ = 1,825, *p* < 0.003], and serenity [*t*_(1492)_ = 4,964, *p* < 0.001], as seen in.

Moreover, although men are more optimistic, they are less committed to countering the pandemic than women through preventive measures. The chi-square test showed a significant relationship between optimism and gender, and between commitment and gender, respectively χ^2^ (3, 1492) = 22.7, *p* < 0.001 and χ^2^ (3, 1492) = 23.885, *p* < 0.001.

**Figure 4:**
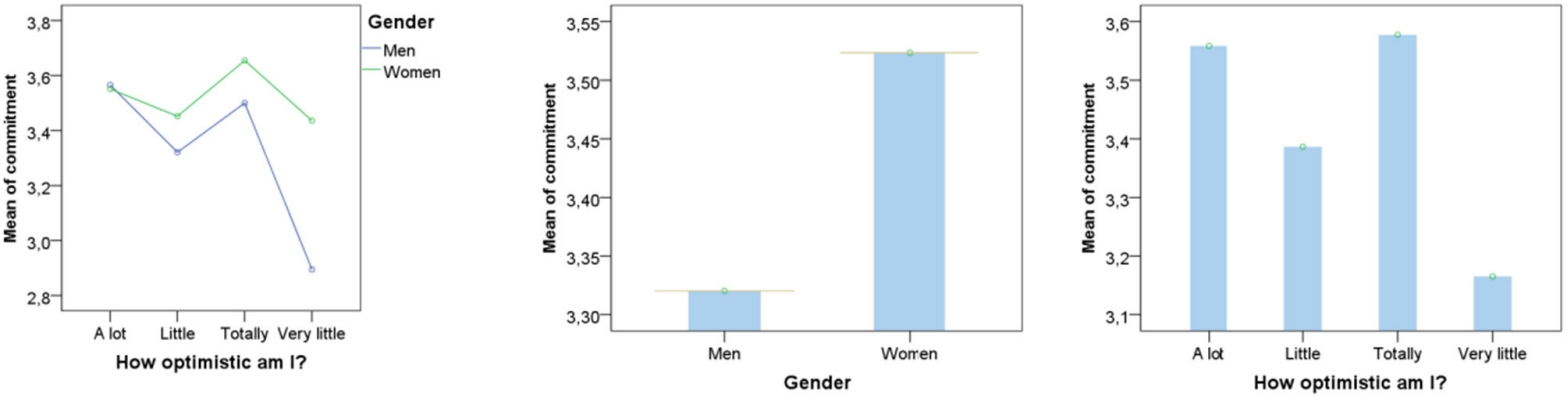
Women are committed to counter the pandemic, although they are less optimistic.

An ANOVA was achieved to check if men and women are significantly different regarding their optimism and commitment. Optimism, gender, and their interaction had a significant effect on commitment, respectively, *F*_(3, 1489)_ = 9.427, *p* < 0.001, η^2^ = 0.19; *F*_(1, 1491)_ = 11.939, *p* < 0.001, η^2^ = 0.18; *F*_(3, 1489)_ = 3.341, *p* < 0.02, η^2^ = 0.17 (Figure 4).

### Age Effect: Elders and 35–45 Years Old Age Group Are More Aware and Prudent

The perception and evaluation of the dangerousness of the pandemic depend on the age of the respondent, since the older the respondent, the closer his evaluation is to reality: χ^2^ (6, 1492) = 23.435, *p* < 0.001.

The most careful age group regarding the preventive behavior “do not touch others” and “keep the safety distance of one m” is 35–45 years, respectively χ^2^ (9, 1492) = 57.337, *p* < 0.001, and χ^2^ (9, 1492) = 56.024, *p* < 0.001.

Although the 35–45 age group is the most cautious and the most committed to countering the pandemic, it is the one who especially feels fear, χ^2^ (9, 1492) = 32.445, *p* < 0.0001.

The 20–35 and 35–45 age groups are the age groups who especially expressed sadness, χ^2^ (9, 1492) = 25.843, *p* < 0.003.

The youngest age group (<20 years) is the most optimistic group, χ^2^ (9, 1492) = 17.602, *p* < = 0.05.

### Interconnections Between Awareness, Attitude, and Behavior

Respondents who described the pandemic as very dangerous responded better to “lockdown in case of doubt” and seemed to take the necessary preventive behaviors (hand cleaning, keeping the social distancing) and are more committed to countering the pandemic and to changing their behaviors. Indeed, the chi-square test of independence showed a significant relationship between the perception of the pandemic dangerousness and the lockdown acceptance in case of doubt: χ^2^ (3, 1492) = 45.59, *p* < 0.001.

The lockdown acceptance in case of doubt was positively related to the preventive behaviors, “cleaning hands” [χ^2^ (3, 1492) = 108.08, *p* < 0.0001], “Do not touching others” [χ^2^ (3, 1492) = 43.031, *p* < 0.0001], “Social distancing” [χ^2^ (3, 1492) = 54.01, *p* < 0.0001].

The relationship between the awareness of the dangerousness of the pandemic and the commitment to counter the pandemic is significant: χ^2^ (3, 1492) = 37.399, *p* < 0.0001.

In the same vein, the lockdown acceptance in all cases depends significantly on the awareness of the dangerousness of the pandemic [χ^2^ (3, 1492) = 53.017, *p* < 0.0001], and the evaluation/awareness of its speed of spread [χ^2^ (3, 1492) = 15.862, *p* < 0.001].

Accepting lockdown in all cases was positively related to the preventive behaviors: “Hand cleaning” [χ^2^ (3, 1492) = 44.604, *p* < 0.0001], “do not touching others” [χ^2^ (3, 1492) = 64.56, *p* < 0.0001], and social distancing [χ^2^ (3, 1492) = 107.95, *p* < 0.0001].

In fact, the more the respondent is aware of the dangerousness of the pandemic and its speed of spread, the more he is prepared to accept lockdown and to take the necessary precautions behaviors (cleaning hands, keeping social distancing, do not touch the others); he is also more willing to counter the pandemic and to change his behavior. The positive relationship between the awareness of the dangerousness of the pandemic and the readiness to change behavior was significant: χ^2^ (9, 1492) = 273,752, *p* < 0.0001.

## Discussion

Public health issues, such as the COVID-19 pandemic, have been shown in studies to trigger psychological issues in people, with symptoms including anger, fear, anxiety, hopelessness, and boredom (5, 7, 8). This survey-based study was conducted during the outbreak of the COVID-19 pandemic and had 1,492 participants; we examined public awareness, emotion, and motivation toward COVID-19 and evaluated the influence of these factors on the commitment and behavior of people.

This study aimed to find the relationship between covid-19 and the people's psychological effects, emotions, and behavior, crossed with the influence of age and gender. We found a significant relationship between gender and acceptance of the lockdown. Women are significantly more convinced about Lockdown and Lockdown in case of doubt about their infection than men. This finding corroborates the results obtained by Brooks and his team (28) in their study of people's behavior and reaction to the quarantine.

It can also be observed that women are more aware of the dangerousness of the pandemic as a significant relationship between gender and awareness of the dangerousness of the pandemic was observed. The results also showed that women, in comparison to men, are more cautious when interacting with the world. Women follow more precautions in handwashing, social distancing, and mask-wearing to minimize the transmission of the coronavirus. Gender has a significant impact on perceiving the risks and coping strategies (29).

It has been found that men are more likely to be affected by the COVID-19 outbreak in terms of mortality rate compared to women since the men's and women's chromosomes, genes, and hormones are released to show different responses against viral infections. It has been observed that women are showing more severe responses against COVID-19 and complying with the restraining measures. From the research, it has been suggested that both men and women were initially at normal status. However, then women were paying more attention and suffering from high anxiety levels compared to men. They started to wear hand gloves and surgical masks. Women experienced more psychological problems during the pandemic than men, which highly affected their performance and health conditions (47).

When the effect of COVID-19 was seen regarding emotion, motivation, and commitment according to gender, most female respondents were seen to be more mentally exhausted and more likely than men to feel fear and sadness. This finding corresponds to results obtained by Broche-Pérez and his team (38), who looked at gender differences in COVID-19 fear and concluded that women became more psychologically vulnerable during the epidemic and that gender was a major predictor of COVID-19 fear. The WHO had previously stated that the pandemic should be viewed from a gender perspective because it affects men and women differently. Women tend to be the most affected by the current pandemic, with rates of gender-based violence increasing during quarantine (34). Although men are more optimistic, they are less committed to countering the pandemic than women through preventive measures. It can be perceived that men and women are considerably different regarding their optimism and commitment. This study demonstrated that women are committed to counter the pandemic, although they are less optimistic (47, 52). The severity perception of the pandemic was divided among the respondent's age group. When the respondents were divided according to age group, it can be observed that the older participants were closer to reality than the younger people. The results displayed that the most cautious regarding “keeping the distance with others of one meter” and “no touching rule” was 35–45 years old. Even though this age group is the most careful, it is most committed to countering the pandemic and the one who feels mostly fear. In terms of sadness, the 20–35 and 35–45 age groups are the most age groups who expressed dismay and lack of positive emotions, while the youngest age group (<20) was the most optimistic regarding the whole COVID-19 situation. When it comes to controlling both risky behaviors in implementing precautionary measures and broad individual reactions to COVID-19 contexts, optimism's defensive position opens up intriguing possibilities for counterbalancing positively and negatively affect (53).

Our imagination may not be reliable when drawing assumptions about the future, especially when it comes to health issues, which is why health-related interaction must be explicit about the risks, but a positive attitude may be adaptive in overcoming hardships. Indeed, positive messages reverberated worldwide: All will be fine, then everything will be fine (53–56).

The results prove a direct relationship between perception and general commitment; however, the indirect effect of perception on the general commitment can also be seen. Perception affects commitment which in turn affects the general commitment. Also, there is another indirect relationship as the perception affects the commitment that affects the emotion and directly affects the general commitment. Perception also has a direct connection with emotion and commitment. So, it can be deduced that perception plays a vital role in determining and involving the other variables.

Furthermore, there was a direct effect of perception on the two variables (evaluation and speed). At the same time, emotion had a direct relationship with the five emotional variables (as anger, fear, disgust, contempt, and sadness), which shows that they had a positive association with each other. The general commitment is directly associated with people's communication and behavior, proving that the lower level of public commitment affects the poor behavior and communication among people amid this pandemic. At the same time, the commitment had a direct association with the hand cleaning, no-touch, and the social behavior of people.

Higher potential risk and severity of transmitting the novel coronavirus, higher perceived comparative susceptibility and harm to the body from SARS, and more uncertainty about knowledge reliability, according to the study, were all significantly and positively correlated with recorded medium risk and intensity of catching the novel coronavirus. On the other hand, strong self-confidence was positively linked to a lower risk during the epidemic (40).

## Conclusion

In this study, we compared the effect of COVID-19 on the awareness, emotion, and motivation of the Tunisians. We found that gender disparity can be seen in how COVID-19 risk perception can be observed. Women, in general, are more cautious about the coronavirus when interacting with the world. Higher precautions are taken by women in hand cleaning, social distancing, and covering face with the mask. Women's psychological and emotional state is more toward the sadness and pessimistic side than men who are more carefree about the whole situation. Compared with the age, the older age group is more serious about the Covid-19 pandemic in countering it. The younger age group seems to be more optimistic about the situation. The more the respondent is aware of the seriousness of the pandemic and its speed of spread, the more he is prepared to accept lockdown and to take the necessary precautions (cleaning hands, keeping social distancing, do not touch the others) and more his emotions of fear, contempt, disgust, sadness are strong. He is also more willing to counter the pandemic and to change his behavior. The younger age group has a lower mortality rate compared to the adults. Seeking effective coping mechanisms is imperative.

An effective communication system must be achieved during COVID-19 (i.e., content, method, people, and partners). Content is considered phased and situation-specific, ensuring the communication precedes and monitors the operational and community response during the outbreak. In the process, different platforms should be included. For instance, blogs, call centers, webinars, conference calls, online health group videos, digital news media are the means to ensure communication. First, it is highly recommended to make a gender-sensitive communication that appeases their negative emotions and transform their commitment into a powerful mean to engage the overall society and to increase the men's commitment. It is also recommended to help youngers positively so that they can express their feelings. On the other hand, engage older adults in other safe things (as they become more aggressive and emotionally depressed) that will make them feel relaxed, loving daily exercise, regular sleep schedules, and eating nutritious food is all recommended. Emotions play an important role when dealing with any crisis. In the time of the pandemic, it is highly recommended to stay positive and optimistic. The WHO and its agencies in the different countries should be the leader of the psychological management of the pandemic crisis, based on studies like the present one. Social media is the biggest platform for any awareness program. Hence, awareness of the sanitary methods in the people can be done through various social media platforms. Finally, policymakers should be more aware and practical regarding the psychological management of the pandemic and any kind of new health crisis.

## Data Availability Statement

The raw data supporting the conclusions of this article will be made available by the authors, without undue reservation.

## Ethics Statement

For this study, ethical approval has not been necessary since the data are properly anonymized and informed consent was obtained at the time of original data collection.

## Author Contributions

The author confirms being the sole contributor of this work and has approved it for publication.

## Conflict of Interest

The author declares that the research was conducted in the absence of any commercial or financial relationships that could be construed as a potential conflict of interest.

## Publisher's Note

All claims expressed in this article are solely those of the authors and do not necessarily represent those of their affiliated organizations, or those of the publisher, the editors and the reviewers. Any product that may be evaluated in this article, or claim that may be made by its manufacturer, is not guaranteed or endorsed by the publisher.
