# Supplementary material for: How Did Awareness, Emotion, and Motivation Shape Behavior Toward COVID-19 in Tunisians?
Source: Front Public Health. 2021 Dec 31;9:771686. doi: 10.3389/fpubh.2021.771686 (PMC8759456; doi:10.3389/fpubh.2021.771686)
Supplement: Supplementary file 1 [file Presentation_1.pdf]

How did awareness, emotion, and motivation shape behavior toward COVID-19 in Tunisians?

Presentation

Plan

Introduction

Objective

Method

Results

Conclusion

Introduction

- COVID-19's unpredictability and ambiguity endanger not only people's physical health but also their mental well-being, especially in terms of emotions and comprehension, according to several studies.

- People are more likely to experience negative emotions (e.g., irrational fear, anxiety, etc.) and negative cognitive appraisal for self-protection.

- As a result, it's important to grasp the potential psychological effects of COVID-19 as quickly and safely as possible.

Objective

- Therefore, this study was conducted to assess the psychological distress in the general population of Tunisia during the epidemic of COVID-19 and to examine how awareness of the disease, emotional experience and motivation shaped the behavior toward COVID-19.

Method

- The study was conducted to evaluate the psychological impacts of the COVID-19 pandemic in the Tunisian population. The sample of the study is composed of 1492 Tunisian participants.
- Gender effects and age limits were studied in relation with the seriousness of the disease and the lockdown impacts.
- The data was analyzed and interpreted using the chi-square test, path analysis, and confirmatory factor analysis. The study also examined the effects of COVID-19 regarding emotion, motivation, and commitment according to gender.

Results

- We found a significant relationship between gender and awareness about the lockdown. Women are significantly more convinced about lockdown and lockdown in case of doubt about their infection than men.

- It can also be perceived that women are more aware of the dangerousness of the pandemic as a significant relationship between gender and awareness of the dangerousness of the pandemic was observed.

Results

- Although men are more optimistic, they are less committed to countering the pandemic than women through preventive measures.
- It can be perceived that men and women are considerably different regarding their optimism and commitment.
- This study demonstrated that women are committed to counter the pandemic, although they are less optimistic.

Results

- In terms of sadness, the 20-35 and 35-45 age groups are the most age groups who expressed dismay and lack of motivation, while the youngest age group (less than 20) were the most optimistic regarding the whole Covid-19 situation.

Conclusion

- The more the respondent is aware of the seriousness of the pandemic and its speed of spread, the more he is prepared to accept lockdown and to take the necessary precautions (cleaning hands, keeping physical distancing, do not touch the others) and more his emotions of fear, contempt, disgust, sadness are strong.
- An effective communication system must be achieved during COVID-19. Content is considered phased and situation-specific, ensuring the communication precedes and monitors the operational and community response during the outbreak.

Conclusion

- Finally, policymakers should be more aware and practical regarding the psychological management of the pandemic.

References

Azlan, A. A., Hamzah, M. R., Sern, T. J., Ayub, S. H., & Mohamad, E. (2020). Public knowledge, attitudes and practices towards COVID-19: A cross-sectional study in Malaysia. Plos One, 15(5), e0233668.

Bao, Y., Sun, Y., Meng, S., Shi, J., & Lu, L. (2020). 2019-nCoV epidemic: address mental health care to empower society. The Lancet, 395(10224), e37–e38.

Ferguson, N. (2007). Capturing human behaviour. Nature, 446(7137), 733.

Group, W. H. O. W. (2006). Nonpharmaceutical interventions for pandemic influenza, national and community measures. Emerging Infectious Diseases, 12(1), 88.

Houston, V., & Bull, R. (1994). Do people avoid sitting next to someone who is facially disfigured? European Journal of Social Psychology, 24(2), 279–284.

References

Kiecolt-Glaser, J. K., McGuire, L., Robles, T. F., & Glaser, R. (2002). Emotions, morbidity, and mortality: New perspectives from psychoneuroimmunology. *Annual Review of Psychology*, *53*(1), 83–107.

Mortensen, C. R., Becker, D. V., Ackerman, J. M., Neuberg, S. L., & Kenrick, D. T. (2010). Infection breeds reticence: The effects of disease salience on self-perceptions of personality and behavioral avoidance tendencies. *Psychological Science*, *21*(3), 440–447.

Norris, F. H., Friedman, M. J., & Watson, P. J. (2002). 60,000 disaster victims speak: Part II. Summary and implications of the disaster mental health research. *Psychiatry: Interpersonal and Biological Processes*, *65*(3), 240–260.

Qiu, J., Shen, B., Zhao, M., Wang, Z., Xie, B., & Xu, Y. (2020). A nationwide survey of psychological distress among Chinese people in the COVID-19 epidemic: implications and policy recommendations. *General Psychiatry*, *33*(2).
