# Supplementary material for: How Did Awareness, Emotion, and Motivation Shape Behavior Toward COVID-19 in Tunisians?
Source: Front Public Health. 2021 Dec 31;9:771686. doi: 10.3389/fpubh.2021.771686 (PMC8759456; doi:10.3389/fpubh.2021.771686)
Supplement: Supplementary file 2 [file Presentation_2.pdf]

APPENDIX

AB SURVEY COVID-19 – Slim Masmoudi, Ph.D

My attitude and my behaviors towards the Coronavirus pandemic

Mon attitude et mes comportements face à la pandémie Coronavirus

موقفي وسلوكي تجاه وباء كورونا

Demographic section

Q1

My gender - Mon genre - نوعي

Man - Homme - رجل

Woman - Femme - امرأة

Q2

My birthday - Ma date de naissance - تاريخ ميلادي

Month, day, year - Mois, jour, année

Q3

My age - Mon âge - عمري

Q4

My instruction level - Mon niveau d'instruction - المستوى التعليمي

1. Primary school - Enseignement primaire - تعليم ابتدائي
2. Secondar school - Enseignement secondaire - تعليم ثانوي
3. High school diploma - Baccalauréat - بكالوريا
4. Bachelor's degree - Licence - إجازة
5. Master - ماجستير
6. PhD - Doctorat - دكتوراه

Q5

My governorate - Mon gouvernorat - ولايتي

1. Ariana - أريانة
2. Béja - باجة
3. Ben Arous - بن عروس

4. Bizerte - بنزرت

5. Gabès - قابس

6. Gafsa - قفصة

7. Jendouba - جندوبة

8. Kairouan - القيروان

9. Kasserine - القصرين

10. Kébili - قبلي

11. Le Kef - الكاف

12. Mahdia - المهدية

13. La Manouba - منوبة

14. Médenine - مدنين

15. Monastir - المنستير

16. Nabeul - نابل

17. Sfax - صفاقس

18. Sidi Bouzid - سيدي بوزيد

19. Siliana - سليانة

20. Sousse - سوسة

21. Tataouine - تطاوين

22. Tozeur - توزر

23. Tunis - تونس

24. Zaghouan - زغوان

Awareness/Perception section

Q6

How do I assess the severity of the pandemic? - Comment j'évalue la gravité de la pandémie ? - كيف أقيّم خطورة الوباء

Not at all dangerous - Pas du tout dangereux - ليس خطيرا بتاتا

A little dangerous - Un peu dangereux - خطير قليلا

Dangerous - Dangereux - خطير

Very dangerous - Très dangereux - خطير جدا

Q7

Speed of spread - Vitesse de propagation - سرعة تفشي العدوى

It spreads very slowly - Il se propage très lentement - يتفشّى ببطء كبير

It spreads slowly - Il se propage lentement - يتفشّى ببطء

It spreads quite quickly - Il se propage assez rapidement - يتفشّى بسرعة كافية

It spreads very quickly - Il se propage très rapidement - يتفشّى بسرعة كبيرة

Commitment/Behavior section

Q8

To what extent do I respect these preventive behaviors? - A quel point je respecte ces comportements préventifs ? - إلى أي حد أحترم السلوكات الوقائية التالية؟

Q8.1. Hands cleaning - Nettoyer les mains - تنظيف اليدين

Very little - Très peu - قليلا جدا

Little - Peu – قليلا

Often - Souvent – غالبا

Always - Toujours - دائما

Q8.2. Don’t touch others - Ne pas toucher les autres - لا ألمس الآخرين

Very little - Très peu - قليلا جدا

Little - Peu – قليلا

Often - Souvent – غالبا

Always - Toujours - دائما

Q8.3. Keep a distance of one meter from others Garder la distance d'un mètre des autres - أبقى على مسافة متر من الآخرين

Very little - Très peu - قليلا جدا

Little - Peu – قليلا

Often - Souvent – غالبا

Always - Toujours - دائما

Q9

I do my self-isolation when in doubt - Je fais mon auto-isolation en cas de doute - أنفذ الحجر الصحي التلقائي في حالة الشك في العدوى

Yes - Oui - نعم

No - Non - لا

Q10

I do my 14-day self-isolation in any case - Je fais mon auto-isolation de 14 jours dans tous les cas - أنفذ الحجر الصحي التلقائي لمدة 14 يوما في كل الحالات

Yes - Oui - نعم

No - Non - لا

Emotion section

Q11

How do I feel about the current pandemic situation and to what degree? - Quelles sont mes émotions face à la situation pandémique actuelle et à quel degré - ماهي انفعالاتي تجاه الوضعية الوبائية الحالية وبأية درجة

1 2 3 4

Q11.1. Anger - Colère - الغضب

Q11.2. Fear - Peur - الخوف

Q11.3. Joy - Joie - الفرح

Q11.4. Disgust - Dégoût - التقزز

Q11.5. Contempt - Mépris - الازدراء

Q11.6. Sadness - Tristesse - الحزن

Q11.7. Serenity - Sérénité - الصفاء

Motivation/General Commitment

Q12

How committed am I to the fight against the pandemic with my country? - A quel point je m'engage avec mon pays dans la lutte contre la pandémie ? - إلى أي حد ألتزم مع بلادي لمقاومة الوباء ؟

Very little - Très peu - قليلا جدا

Little - Peu - قليلا

Much - Beaucoup - كثيرا

Totally - Totalement - بصفة مطلقة

Q13

How much have I changed my behavior in fighting the pandemic? - A quel point j'ai changé mon comportement dans la lutte contre la pandémie ? - إلى أي حد غيرت سلوكي لمقاومة الوباء ؟

Very little - Très peu - قليلا جدا

Little - Peu - قليلا

Much - Beaucoup - كثيرا

Totally - Totalement - بصفة مطلقة

Q14

To what extent am I optimistic ? - A quel point je suis optimiste ? - إلى أي حد أنا متفائل ؟

Very little - Très peu - قليلا جدا

Little - Peu - قليلا

Much - Beaucoup - كثيرا

Totally - Totalement - بصفة مطلقة
